# Hugo RAS Versus Da Vinci in Colorectal Surgery: A Systematic Review and Outcome Meta-Analysis

**DOI:** 10.3390/jcm15176736

**Published:** 2026-08-30

**Authors:** Sascha Vaghiri, Dimitrios Prassas, Emmanuella Habib, Norma Schmitz-Conti, Wolfram Trudo Knoefel, Andrea Alexander

**Affiliations:** 1 Department of Surgery (A), Heinrich-Heine-University, Medical Faculty and University Hospital Duesseldorf, Moorenstr. 5, 40225 Duesseldorf, Germany; sascha.vaghiri@med.uni-duesseldorf.de (S.V.); dimitrios.prassas@med.uni-duesseldorf.de (D.P.); emmanuella.habib@med.uni-duesseldorf.de (E.H.); knoefel@med.uni-duesseldorf.de (W.T.K.); andrea.alexander@med.uni-duesseldorf.de (A.A.); 2 Department of Surgery, Katholisches Klinikum Essen, Philippusstift, Teaching Hospital of Duisburg-Essen University, Huelsmannstr, 17, 45355 Essen, Germany

**Keywords:** robotic surgery, Hugo RAS, Da Vinci, perioperative outcomes

## Abstract

**Background:** The advantages of robotic surgery have increasingly led to its rapid adoption in surgical practice. In this context, new robotic platforms such as Hugo RAS have been introduced in recent years to meet the demand for cost-effective alternatives to the well-established Da Vinci system. However, high-quality evidence directly comparing the operative outcomes of these platforms remains scarce. **Methods:** The current PRISMA and Cochrane guidelines were followed. After careful study selection using the MEDLINE, Scopus, Google Scholar, and Cochrane Register databases, relevant operative and oncologic outcomes were extracted and analyzed from suitable studies comparing the Hugo RAS and Da Vinci platforms in colorectal surgery. ROBINS-I and GRADE were applied to judge the risk of bias and evidence level, respectively. **Results:** Five comparative studies with a total of 370 patients (Hugo RAS: n = 130; Da Vinci: n = 240) were included in the final analysis. Operative duration (min) was significantly longer in the Hugo RAS cohort than in Da Vinci procedures (MD 25.98, 95% CI: 6.35–4.62; *p* = 0.009; I^2^ = 38%). Other endpoints, including the duration of hospitalization, operative morbidity, and oncological parameters, were comparable between the groups, although considerable heterogeneity was observed for length of hospital stay. The amount of blood loss (mL) was reported to be lower in the Da Vinci group in four studies, whereas one study found lower blood loss in the Hugo RAS group, resulting in conflicting findings and substantial heterogeneity across the included studies. The overall risk of bias ranged from moderate to serious. **Conclusions:** The results presented here suggest that colorectal surgery using the modular Hugo RAS platform is technically reliable and effective with apparently comparable perioperative outcomes to the Da Vinci system. Nevertheless, serious methodological and statistical limitations, as well as inter-study heterogeneity, warrant further high-quality, standardized, multi-institutional studies.

## 1. Introduction

Minimally invasive surgery has gained an established role in the surgical management of benign and malignant colorectal diseases over recent decades. The laparoscopic technique has consistently shown several benefits in comparison to open surgery, including reduced postoperative pain, faster rehabilitation, and hospital discharge, while achieving similar oncological outcomes [1,2]. Since its clinical introduction, the Da Vinci (Intuitive Surgical, Sunnyvale, CA, USA) surgical system (with the current fifth-generation) has undoubtedly changed and revolutionized the field of colorectal surgery. Compared with conventional laparoscopy, the platform offers several technical advantages, including three-dimensional visualization, tremor filtration, enhanced dexterity, and superior ergonomic design, all of which have contributed to improved surgical precision and performance [3]. These features facilitate the performance of complex procedures, particularly in anatomically confined areas such as the pelvis [4,5,6,7]. However, limited accessibility in some parts of the world, as well as high acquisition and maintenance costs, partly due to Intuitive’s monopoly position in the surgical robotic market, have led to the development of new, innovative, and more cost-effective robotic systems [8,9,10]. The Hugo™ Robotic-Assisted Surgery (RAS) system (Medtronic, Minneapolis, MN, USA) was presented in 2019 as a next-generation modular robotic platform with decentralized robotic arms, an open-console design, and a high-definition three-dimensional visualization system. This conceptual design and configuration of Hugo RAS, in contrast to the boom-mounted Da Vinci system, allow flexible and patient-tailored port placement and cart adjustment inside the operating room (OR), while improving the surgeon’s ergonomics, situational awareness, and interaction with the surgical team [11,12,13,14].

The Hugo RAS platform obtained CE marking for general surgery in October 2022, and two Italian studies subsequently reported its inaugural use in colon and rectal surgery [15,16]. Since then, various colorectal surgery institutions have implemented this new surgical robot in their daily clinical practice [12,17,18]. Furthermore, the Hugo RAS platform has been increasingly adopted in urology [19,20], general surgery [21,22], and gynecology [23] disciplines. Despite consistently promising results regarding the technical performance and clinical reliability of the Hugo RAS platform in colorectal surgery [24,25], comparative outcome and performance studies of the Hugo RAS and Da Vinci robotic systems remain scarce. At the same time, the strength of the current literature is limited by small sample sizes, heterogeneous patient cohorts, different types of procedures, and varying levels of surgeon experience [14,26,27].

To date, no comprehensive review and meta-analysis has specifically analyzed the existing comparative evidence for the Hugo RAS and Da Vinci robotic platforms in colorectal surgery. Therefore, the primary objective was to provide a robust synthesis of the pooled operative outcomes and oncological results in colorectal procedures using the Hugo RAS and Da Vinci robotic platforms.

## 2. Materials and Methods

This systematic review and meta-analysis was performed according to the PRISMA (Supplementary File S1; Preferred Reporting Items for Systematic Reviews and Meta-Analyses) and Cochrane Handbook for Systematic Reviews guidelines [28,29]. The PROSPERO registration ID was obtained (CRD420261457400).

### 2.1. Literature Search

Two authors (S.V. and D.P.) initially screened titles and abstracts in PubMed (MEDLINE), the Cochrane Central Register of Controlled Trials (CENTRAL), Scopus, and Google Scholar. Suitable articles were then further evaluated by full-text review. All comparative studies analyzing intra- and postoperative outcomes of the Hugo RAS versus Da Vinci robotic platforms in colorectal surgery were eligible. The following medical keywords were combined: [(colorectal OR colectomy) AND (Hugo RAS OR Da Vinci OR robotic OR robotic-assisted)]. The most recent literature review was conducted on 10 June 2026 and was limited to English-language papers but with no time limitations. In addition to the full-text review, the reference lists of all retrieved sources were comprehensively evaluated for identifying further eligible sources. All authors agreed on the final decision regarding which studies to include at this stage.

### 2.2. Eligibility Criteria

Eligibility criteria were defined based on PICO:

- P (Population): Patients undergoing colorectal surgery for benign and/or malignant indications using a fully robotic platform.
- I (Intervention): Procedures performed using the Da Vinci robot.
- C (Comparison): Colorectal surgery performed using the Hugo RAS platform.
- O (Outcomes): Duration of surgery (min) as the primary outcome, and intraoperative complications, estimated blood loss (mL), postoperative complications and major morbidity (according to the Clavien–Dindo classification [30]), redo surgery and readmission rates, length of hospital stay (LOS), number of harvested lymph nodes, and rate of R0 resection.

Accordingly, all comparative studies of any design reporting perioperative, pathological, and platform-specific outcomes of patients undergoing colorectal surgery with either the Hugo RAS platform or Da Vinci surgical robot were considered eligible. No sample size restriction was imposed for the included studies. Exclusion criteria were as follows: (1) a non-comparative study setting, (2) duplicate articles, (3) non-adult patients (aged < 18 years), and (4) studies comparing robotic platforms other than Hugo RAS and Da Vinci.

### 2.3. Data Extraction

Two authors (S.V. and E.H.) extracted all available and relevant data from eligible studies. Besides the above-mentioned outcomes of interest, the following information was extracted: (a) the date of publication and country where the study was conducted, author names, the recruitment period, study design, and the sample size of each group; (b) patient demographics, tumor characteristics and localization, and administration of neoadjuvant treatment; and (c) the type of resection and which robotic platform used, robotic-specific information, and the number of involved surgeons. During the data extraction process, consensus was reached through the agreement of all authors.

### 2.4. Risk-of-Bias and Certainty-of-Evidence Assessment

The ROBINS-I tool [31] was applied to evaluate the risk of bias in all non-randomized studies, as recommended [29]. Two authors (S.V. and N.S.) assessed each study across seven specific domains grouped into pre-intervention, at-intervention, and post-intervention issues to classify the risk of bias. The GRADE (Grading of Recommendations, Assessment, Development, and Evaluation) criteria [32] were used to assess the evidence level for significant outcomes. Differences in bias and certainty-of-evidence assessments were resolved among the involved authors through discussion or consultation with a third senior author (A.A.).

### 2.5. Statistics

Statistical analysis was conducted using RevMan software (version 5.3). Summary treatment-effect estimates with 95% confidence intervals (CIs) were calculated and presented for outcomes of interest. For dichotomous outcomes, odds ratios (ORs) were used as the effect measure, whereas standardized mean differences (SMDs) were calculated to summarize continuous parameters. Where necessary, medians and (interquartile) ranges were converted into means and standard deviations using the methods proposed by Hozo et al. and Luo et al. [33,34]. Fixed- or random-effects models were applied as appropriate. A heterogeneity level of I^2^ > 50% was considered substantial [29] and prompted a leave-one-out sensitivity analysis. A narrative review was provided for outcomes for which quantitative synthesis was not possible. Publication bias tests were not performed as only five studies were included. A *p*-value < 0.05 was the threshold for statistical significance.

## 3. Results

### 3.1. Literature Search

**Figure 1:**
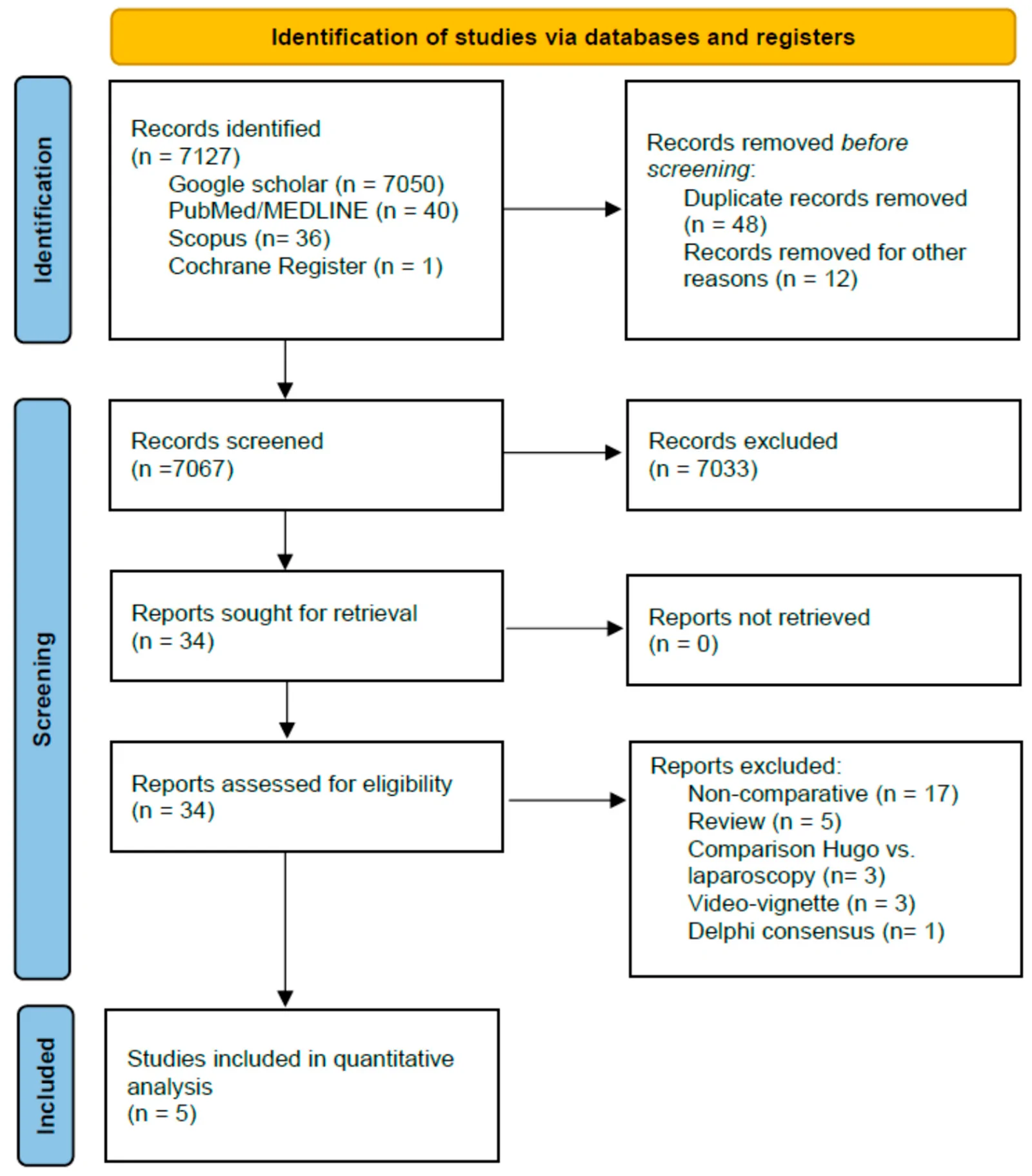
PRISMA flowchart.

The systematic literature search conducted through the defined databases and registries yielded 7127 results. After reviewing all retrieved articles according to our eligibility criteria, a total of five studies were included in the analysis. The detailed literature screening and selection workflow used is depicted in Figure 1.

### 3.2. Study and Cohort Characteristics

A total of 370 patients underwent robotic colorectal surgery using either the Hugo RAS or Da Vinci platform (Hugo RAS: n = 130; Da Vinci: n = 240). Two studies originated from Italy [26,35] and one originated from Belgium [27], and the other two studies were conducted in Japan [6,36]. All studies except that by Toda et al. [36] were single-center studies. In all studies, surgical proficiency and experience with the Da Vinci surgical robot were evident before the implementation of the new Hugo RAS platform. The recruitment period ranged from September 2019 to December 2025 across all studies. The studies conducted by Pedrazzani et al. [26] and Fioccola et al. [35] also compared the outcomes for the Hugo RAS and Da Vinci robots with a third robotic platform (Versius). The fourth-generation Da Vinci (X) surgical system developed by Intuitive was used in four studies [6,26,27,36], whereas Fioccola et al. [35] did not mention which generation of the Da Vinci robot was applied. Two studies included patients with both benign and malignant colorectal disease in their cohorts [26,27], whereas the two Japanese studies focused exclusively on malignant colorectal diseases including rectal cancer [6,36]. Fioccola et al. [35] did not report on the surgical indication, the location of the disease, or the extent of surgery. Baseline patient demographics including gender, age, BMI (body mass index), ASA score (American Society of Anesthesiologists), and tumor stage were similar between the two robotic cohorts in four studies [6,26,27,36]. In the study by Violante et al. [27] that exclusively performed right colectomies in both groups, three robotic arms were used in the Hugo RAS group. In contrast, Toda et al. [36] and Nonaka et al. [6] applied the four-arm configuration for their Hugo RAS procedures. The fourth-arm cart was placed to the right and caudal to the patient in Toda et al. [36] and to the right and cranial in Nonaka et al. [6]. In-hospital and 30-day follow-up data were provided. The study and patient features and the types of surgery performed are shown in Table 1 and Table 2.

### 3.3. Risk-of-Bias Evaluation

**Figure 2:**
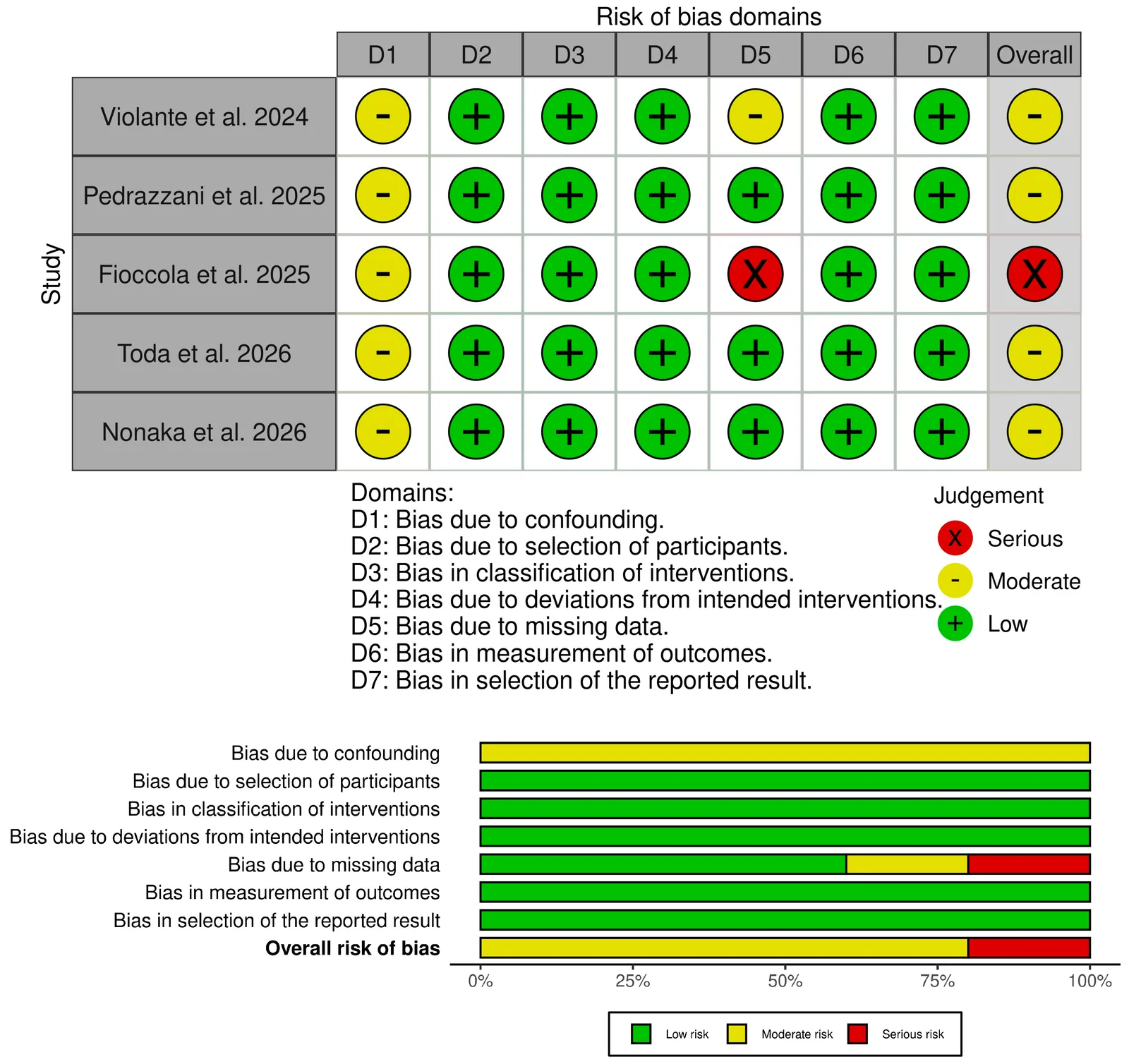
ROBINS-I risk-of-bias summary [6,26,27,35,36].

The overall ROBINS-I risk-of-bias assessment was moderate in four studies [6,26,27,36] and serious in one study [35]. The main methodological limitations in all studies were as follows: (a) non-randomized patient allocation and the retrospective study design; (b) potential selection bias; (c) comparison based on non-contemporaneous cohorts (temporal bias); (d) small sample sizes with a wide range of different procedures; and (e) short follow-up periods. However, baseline patient characteristics were comparable between both robotic cohorts in all studies [6,26,27,35,36]. Propensity score matching (PSM) was performed in two studies [6,36]. The risk-of-bias summary is demonstrated in Figure 2.

### 3.4. Primary Outcome

#### Duration of Surgery

**Figure 3:**
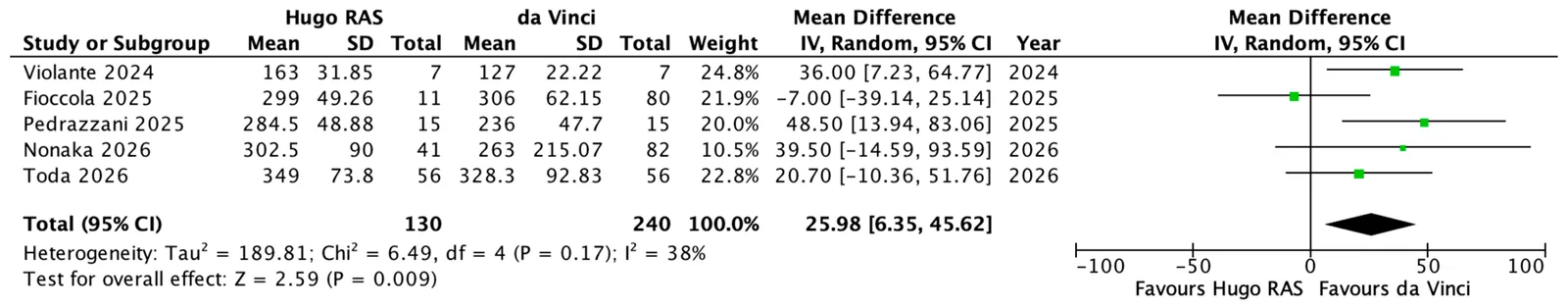
Forest plot, Hugo RAS versus Da Vinci: duration of surgery [6,26,27,35,36].

Surgical duration was reported by all authors [6,26,27,35,36], with 130 patients in the Hugo RAS group and 240 patients in the Da Vinci group, covering a wide range of performed resections. Random-effects meta-analysis demonstrated a significantly longer operative time (by a mean of 25.98 min) with the Hugo RAS robot than with Da Vinci robot (MD 25.98, 95% CI: 6.35–4.62; *p* = 0.009; I^2^ = 38%) (Figure 3). The certainty-of-evidence level was low (Supplementary Table S1).

### 3.5. Secondary Outcomes

#### 3.5.1. Blood Loss

**Figure 4:**
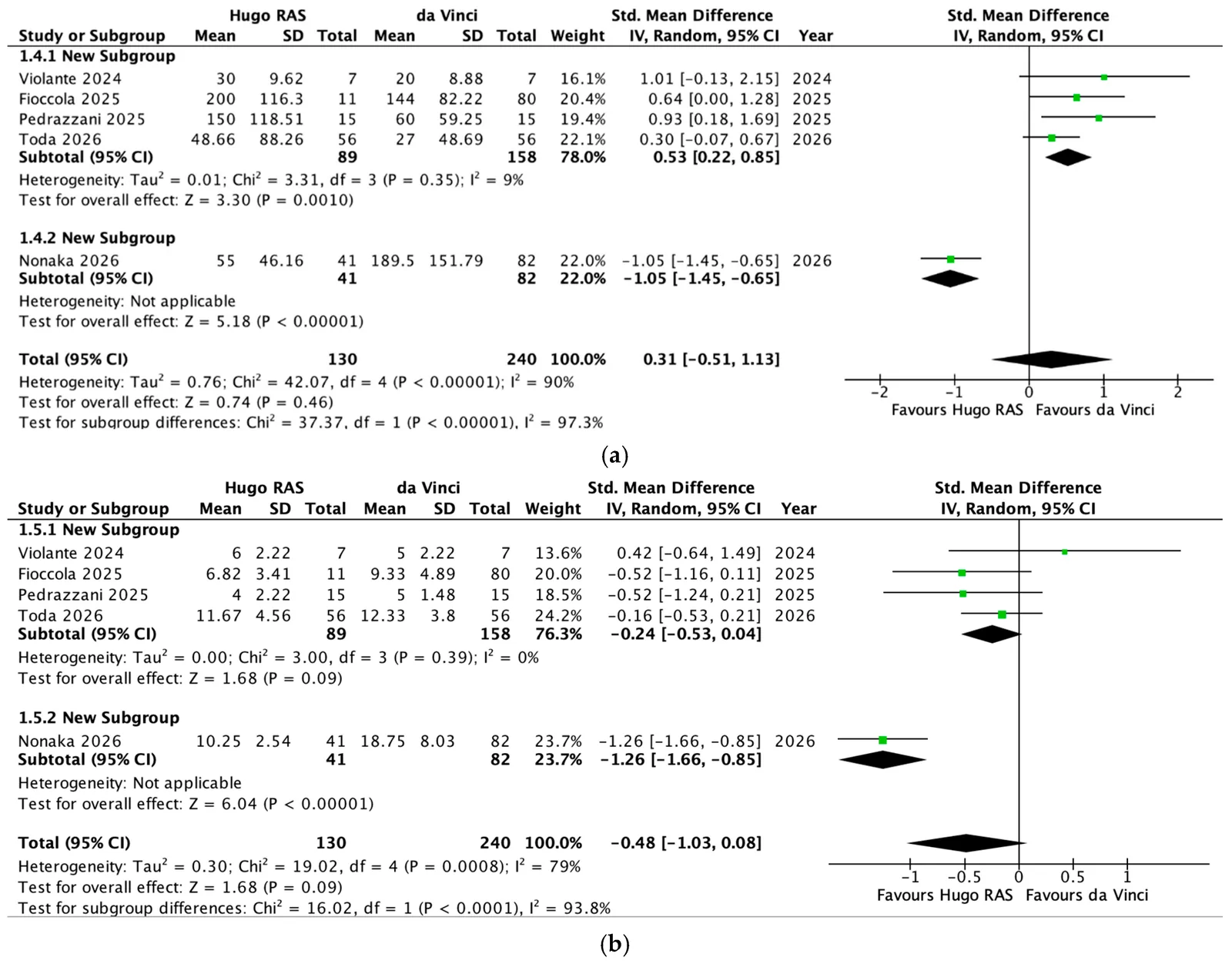

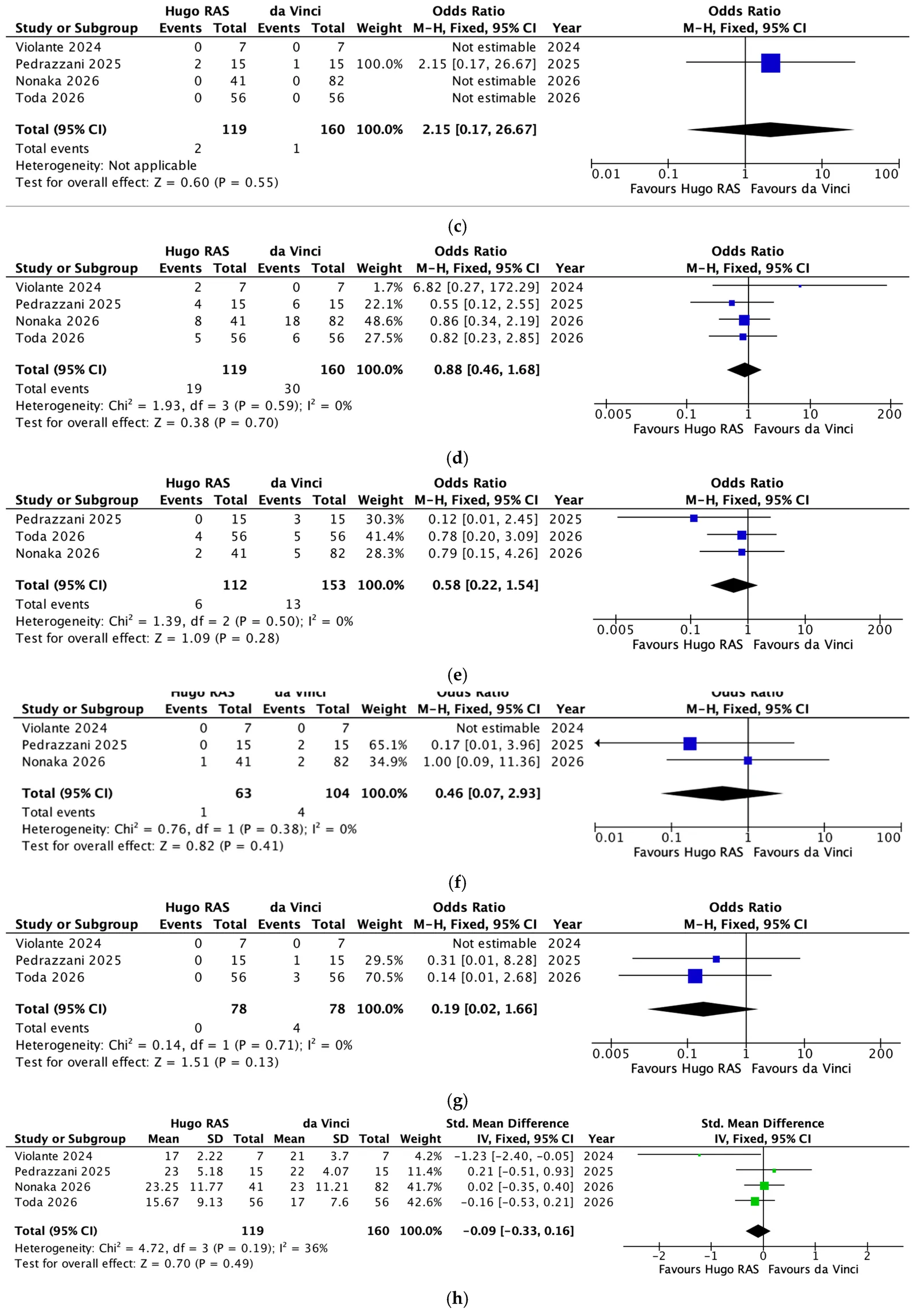

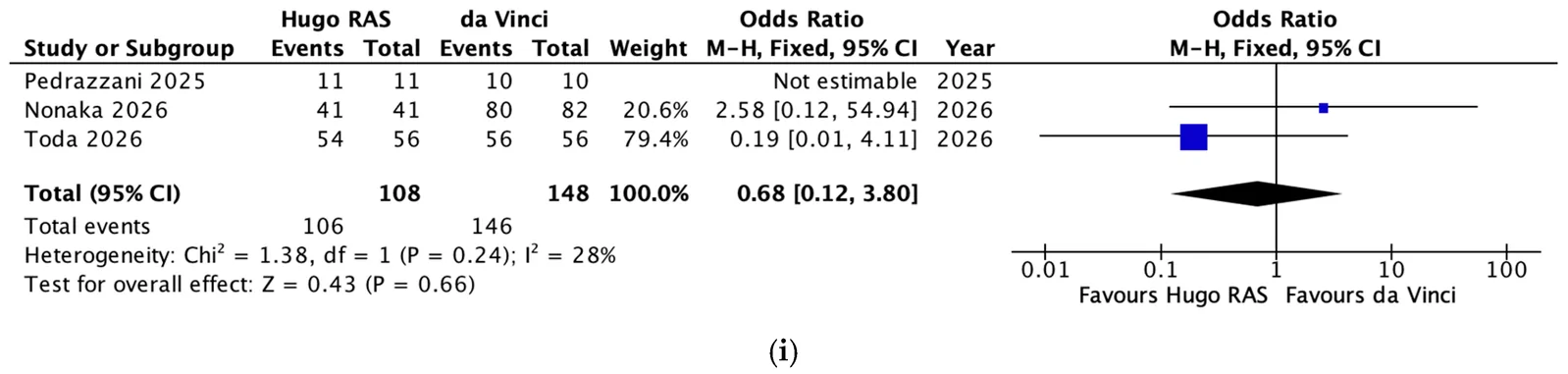
Forest plots, Hugo RAS versus Da Vinci: (**a**) blood loss, (**b**) LOS, (**c**) intraoperative complications, (**d**) overall postoperative complications, (**e**) major complications, (**f**) reoperation, (**g**) readmission, (**h**) the number of harvested lymph nodes, and (**i**) R0 resection [6,26,27,35,36].

Overall blood loss during surgery (mL) was reported in all studies [6,26,27,35,36], comprising a total of 370 patients (Hugo RAS: n = 130, Da Vinci: n = 240). The pooled random-effects analysis demonstrated no significant overall difference in estimated blood loss between Hugo RAS and Da Vinci (SMD 0.31, 95% CI: −0.51 to 1.13; *p* = 0.46). Notably, substantial heterogeneity was observed (I^2^ = 90%). The source of heterogeneity was Nonaka et al. [6], which demonstrated lower blood loss with Hugo RAS (SMD −1.05, 95% CI: −1.45 to −0.65; *p* < 0.00001). In contrast, in the subgroup containing the remaining four studies [26,27,35,36], Hugo RAS procedures were associated with significantly greater blood loss than Da Vinci procedures (SMD 0.53, 95% CI: 0.22–0.85; *p* = 0.001; I^2^ = 9%) (Figure 4a).

#### 3.5.2. Length of Hospital Stay

LOS was recorded in all studies [6,26,27,35,36] involving 130 patients in the Hugo RAS group and 240 patients in the Da Vinci group. Overall, no significant difference was observed in LOS between the Hugo RAS and Da Vinci groups (SMD −0.48, 95% CI: −1.03 to 0.08; *p* = 0.09), although heterogeneity was substantial (I^2^ = 79%). The main contributor to heterogeneity was the study by Nonaka et al. [6], which showed a significantly shorter hospital stay with Hugo RAS (SMD −1.26, 95% CI: −1.66 to −0.85; *p* < 0.00001). In the subgroup of four studies [26,27,35,36] with 89 Hugo RAS patients and 158 Da Vinci patients, no significant difference in LOS was observed between the two study groups (SMD −0.24, 95% CI: −0.53 to 0.04; *p* = 0.09; I^2^ = 0%) (Figure 4b).

#### 3.5.3. Perioperative Complications and Readmission

The rate of perioperative complications was reported in all studies [6,26,27,35,36], involving 370 patients across the Hugo RAS and Da Vinci platforms. The pooled analysis of the reported adverse events indicated no significant differences in the occurrence of intraoperative (OR 2.15, 95% CI: 0.17–26.67; *p* = 0.55) (Figure 4c), overall postoperative (OR 0.88, 95% CI: 0.46–1.68; *p* = 0.70; I^2^ = 0%) (Figure 4d), and major (CD ≥ III) (OR = 0.58, 95% CI: 0.22–1.54; *p* = 0.28; I^2^ = 0%) (Figure 4e) complications between both groups. Regarding reoperation [6,26,27] and readmission rates after surgery [26,27,36], no significant differences were observed in patients undergoing Hugo RAS and Da Vinci procedures (Figure 4f,g).

#### 3.5.4. Oncologic Parameters

A total of four studies [6,26,27,36], comprising 279 patients, were entered in the pooled meta-analysis of harvested lymph nodes (Hugo RAS: n = 119; Da Vinci: n = 160). There was no statistically significant difference in the number of harvested lymph nodes between the two robotic platforms (SMD −0.09, 95% CI: −0.33 to 0.16; *p* = 0.49), with moderate heterogeneity (I^2^ = 36%) (Figure 4h). The R0 resection rate was comparable between the two robotic groups (OR = 0.68, 95% CI: 0.12–3.80; *p* = 0.66; I^2^ = 28%) (Figure 4i) in the three available studies [6,26,36].

### 3.6. Descriptive Outcomes

In the study by Fioccola et al. [35], additional intraoperative variables were analyzed between the Hugo RAS (n = 11) and Da Vinci (n = 80) groups. A significantly steeper Trendelenburg position was required during Da Vinci procedures than in the Hugo RAS cohort (20.2 versus 7.8 degrees; *p* = 0.0001), while other outcomes, such as ventilation parameters, were similar between the two groups. No open conversions were reported in four available studies [6,26,27,36].

## 4. Discussion

This comprehensive analysis represents, to our knowledge, the first quantitative synthesis directly comparing the Hugo RAS and Da Vinci robotic platforms in colon and rectal conditions. The results, encompassing five studies with a total of 370 patients, indicate that in colorectal surgery, the Hugo RAS and Da Vinci platforms seem to have comparable safety and feasibility profiles. However, the largely equivalent perioperative and oncologic outcomes results must be cautiously interpreted because of high risk of type II errors. No significant differences were detected regarding blood loss, length of hospital stay, postoperative morbidity, reoperation rates, readmissions, lymph node harvest, or R0 resection rates. However, in terms of procedural length, we found that the Da Vinci robot was associated with a significantly shorter duration of surgery (by a mean of 25.98 min) compared with the Hugo RAS platform (low certainty-of-evidence level). At first glance, this result is not surprising. The prolonged operative time during Hugo RAS surgery compared with Da Vinci could be explained by several factors, including complex docking maneuvers, trocar configurations, console ergonomics, workflow characteristics, and the platform-specific learning curve during the early phase of adoption and the already achieved competency and experience in the Da Vinci system [26,36,37]. Such factors inevitably prolong operative duration independently of actual surgical performance. Second, the modular-based configuration of Hugo RAS differs substantially from the integrated (boom-mounted) cart design of the Da Vinci system. While the independent four-arm carts offer greater flexibility, multi-quadrant access and potentially improved customization, they may initially require more complex positioning and docking at a specific angle and tilt in the operating room based on the planned procedure [14,15,24]. Several studies have reported that arm docking represents a significant proportion of the additional operative time observed during the early implementation phase of Hugo RAS [18,38,39]. This was further confirmed by Pedrazzani et al. [26] in our analysis, showing that draping, trocar placement, and docking/undocking times were significantly longer with multi-modular robots than with Da Vinci. Additionally, in Nonaka et al. [6], the console time in the Hugo RAS group remained consistently shorter than the total operative time, suggesting that the above-mentioned operative steps may significantly contribute to prolonged procedural length. However, the optimized docking-angle frameworks and multi-docking strategies of the Hugo RAS system have led to reductions in instrument collision and improved access to complex procedures [16,24]. Importantly, this increase in operative duration did not appear to correlate with increased complication rates or inferior surgical quality in colorectal surgery in our analysis.

In a large single-center study with 100 patients undergoing colorectal resections using the Hugo RAS platform, procedure-confined CUSUM analyses demonstrated specific learning curve timelines for different operations (hemicolectomy, anterior resection, and sigmoidectomy) and improvement in operative time as case numbers increased. The global CUSUM analysis revealed learning curve stabilization during the initial phase after approximately 50 cases [25]. Interestingly, all involved surgeons had no prior experience in robotic surgery but did have extensive experience in complex laparoscopic colorectal surgery. In another report from Spain [12], the safety of the Hugo RAS system in colorectal surgery was confirmed by robot-naïve surgeons. Therefore, a structured training program in Hugo RAS appears to be more important than prior robotic proficiency. Indeed, the presence and involvement of technically qualified surgeons in the training process resulted in improved outcomes in robotic surgery [36,40,41]. For the Hugo RAS system, the manufacturer offers the ASCEND program, a structured surgeon-training curriculum across several domains [42]. When interpreting learning curves and operative times for the Hugo RAS system, existing expertise in robotic surgery must be taken into account. In all studies included in this analysis [6,26,27,35,36], surgeons adopting Hugo RAS were highly experienced in performing robotic colorectal surgery with the Da Vinci system. Consequently, the reported learning curve for Hugo RAS does not represent a true robotic learning curve but rather a platform-transition learning curve. Fundamental robotic skills, including three-dimensional visualization, wristed instrumentation, camera control, dissection strategies, and robotic team communication, were already available and well-established. Therefore, the observed equivalence in outcomes might also reflect the transferability of robotic expertise between platforms. On the other hand, the learning process associated with switching between surgical robots should not be underestimated. Although both systems share fundamental robotic principles, important differences exist regarding docking strategies, cart positioning, instrument exchanges, arm configuration, and operating room setup.

Notably, the included colorectal procedures ranged from right colectomies to more complex pelvic resections requiring different robotic setups. Despite the operative time variation resulting from this heterogeneity, the proportions of different procedures appeared to be balanced between the two robotic cohorts across all studies in our analysis. The pooled results demonstrate that blood loss and LOS were comparable between the robotic groups, although substantial heterogeneity was observed. The source of heterogeneity was identified in Nonaka et al. [6]. In that study, the authors implemented a single-docking workflow strategy on the modular Hugo RAS system with phase-dependent instrument allocation and assignment. The greater blood loss and the prolonged hospital stay in the Da Vinci group compared with the Hugo RAS system may be partially attributed to the slightly increased rate of complex anterior and abdominoperineal resections (45% versus 39%) and the changes in perioperative care and surgical approach over the study period. Indeed, Nonaka et al. [6] stated that changing the extraction site during the study from a midline umbilical incision to a Pfannenstiel incision may have contributed to the shorter LOS in the Hugo RAS group by reducing surgical trauma and enhancing postoperative recovery.

In addition to the setup, cart placement, and docking complexity, several other limitations of the Hugo RAS system must be highlighted because some important features and instruments are still not yet fully developed [12]. At present, Hugo RAS has a smaller instrument set than Da Vinci and lacks advanced robotic energy and stapling devices, clip appliers, and an option for indocyanine green fluorescence-guided surgery [14,15,27]. To compensate for this, laparoscopic instruments must be inserted using assistant trocars. Depending on the complexity of the case, several assistant trocars may be necessary. In the study by Pedrazzani et al. [26], almost 50% of patients in the Hugo RAS group required ≥2 additional trocars, while Toda et al. [36] used two assistant trocars as standard. This could lead to higher procurement costs for laparoscopic instruments and increase total incision length, potentially affecting postoperative recovery and pain control. Notably, the performance and efficiency of the Hugo RAS and Da Vinci robotic platforms have been extensively evaluated in urological procedures. The results demonstrated that both platforms have comparable surgical and oncological outcomes [43,44,45].

This meta-analysis has important shortcomings that should be noted when interpreting the findings. First, all included studies had relatively small sample sizes, increasing the potential for selection bias and residual confounding. Furthermore, the limited sample sizes increase the risk of type II errors and may reduce the precision of the pooled effect estimates. Second, substantial heterogeneity exists regarding procedure types, as well as institutional experience and robotic implementation strategies. Performing more granular subgroup analyses according to the type of surgery would have substantially reduced the number of patients in each comparison, thereby further increasing the risk of type II errors.

Third, follow-up durations remain limited, restricting the assessment of long-term oncological and functional outcomes. Finally, many available studies originate from centers with extensive robotic experience, potentially limiting generalizability to lower-volume institutions or surgeons at earlier stages of robotic adoption and the learning curve. Notably, no data regarding procedural costs were provided; therefore, we were unable to perform a pooled analysis of economic aspects of robotic colorectal surgery. Collectively, these confounding factors and the limited statistical power of the pooled analysis preclude demonstrating true clinical equivalence between the two robotic platforms.

## 5. Conclusions

This comprehensive review and meta-analysis demonstrated that the modular Hugo RAS robot is technically reliable and effective. Both platforms achieved comparable perioperative and oncologic outcomes, although operative duration was significantly longer in the Hugo RAS cohort. Furthermore, the reported amount of blood loss (mL) varied considerably across the included studies, precluding robust interpretation.

Given the rapid evolution of robotic surgery, the heterogeneity of the included patient populations and surgical procedures, the lack of statistical power, and the absence of cost-effectiveness analyses, the current evidence remains insufficient to draw definitive conclusions. Prospective multicenter studies with long-term oncological follow-up are therefore needed to establish whether the Hugo RAS system can serve as a reliable alternative to existing robotic platforms.

## Figures and Tables

**Table 1:** Overall study description.

	Publication Year	Origin	Study Design	Study Period	Number of Patients	Indication for Surgery	Robotic Platforms	Number of Surgeons
Violante et al. [27]	2024	Belgium	Retrospective, single-center	NA	14	Benign/malignant	Hugo RAS vs. Da Vinci X	1
Pedrazzani et al. [26]	2025	Italy	Retrospective, single-center (COMPAR-CRC trial)	February–December 2024	30	Benign/malignant	Hugo RAS vs. Da Vinci Xi	2
Fioccola et al. [35]	2025	Italy	Retrospective, single-center	January 2024–June 2025	91 (colorectal surgery)	NA	Hugo RAS vs. Da Vinci	NA
Toda et al. [36]	2026	Japan	Retrospective, bi-center, PSM 1:1	September 2019–October 2025	Overall 211, PSM 112	Malignant (no distant metastases)	Hugo RAS vs. Da Vinci Xi	NA
Nonaka et al. [6]	2026	Japan	Retrospective, single-center, PSM 1:2	April 2020–December 2025	Overall 274, PSM 123	Malignant (no multivisceral resection)	Hugo RAS vs. Da Vinci Xi	Multiple

PSM: Propensity score matching; NA: Not available.

**Table 2:** Summary of patient characteristics, tumor location, and performed resections.

	Groups	Male/Female	Age (Years) *	BMI (kg/m^2^) *	ASA Score	Location Cancer/Pathology	Tumor Stage	Neoadjuvant Therapy	Type of Surgery
Violante et al. [27]	Hugo RAS	3/4	75 ± 13	29.4 ± 9	ASA II 3 ASA III 4	R Colon	NA	NA	R colectomy
	Da Vinci	3/4	77 ± 11.5	25.5 ± 2.9	ASA II 5 ASA III 2	R Colon	NA	NA	R colectomy
Pedrazzani et al. [26]	Hugo RAS	9/6	70 (20)	25.9 (5.3)	ASA I–II 11 ASA III 4	R/L Colon 7/4 Sigmoid 4	clinical I–II 8 III–IV 3	NA	R/L colectomy 7/4 Sigmoidectomy 4
	Da Vinci	4/11	66 (17.5)	24.2 (6.25)	ASA I–II 14 ASA III 1	R/L Colon 7/3 Sigmoid 5	I–II 8 III–IV 2	NA	R/L colectomy 7/3 Sigmoidectomy 5
Fioccola et al. [35]	Hugo RAS	2/9	74.6 ± 14.1 †	25.8 ± 5.9 †	ASA III [II–III] *	NA	NA	NA	NA
	Da Vinci	48/32	68.2 ± 13.3	25.0 ± 4.9	ASA II [II–III]	NA	NA	NA	NA
Toda et al. [36]	Hugo RAS	32/34	61 (52–72)	23.0 (20.6–25.6)	ASA I–II 51 ASA III–IV 5	Rectum Distance AV (cm) 8 (5–12)	I–II 35 III 21	24	AR 44 ISR 3 APR 4 Pelvic exenteration 1
	Da Vinci	36/30	62 (54–72)	22.9 (20.4–24.8)	ASA I–II 52 ASA III–IV 4	Rectum Distance AV (cm) 8 (5–10)	I–II 39 III 16	28	AR 44 ISR 2 APR 4 Pelvic exenteration 0
Nonaka et al. [6]	Hugo RAS	21/20	71 (45–91)	22.2 (15.7–28.2)	ASA I–II 35 ASA III–IV 6	R/T/L Colon 12/1/4 Sigmoid 8 Rectum 16	clinical I–II 33 III–IV 8	1	R/T/L colectomy 12/1/5 Sigmoidectomy 7 AR 16 APR 0
	Da Vinci	41/41	71.5 (35–92)	22.0 (14.3–30.0)	ASA I–II 74 ASA III–IV 8	R/T/L Colon 20/8/8 Sigmoid 8 Rectum 38	I–II 64 III–IV 18	3	R/T/L colectomy 22/2/10 Sigmoidectomy 11 AR 34 APR 3

ISR: Intersphincteric resection; APR: abdominoperineal resection; AR: anterior resection; R Colon: Right colon; L Colon: Left colon; T Colon: Transverse colon; AV: Anal verge; BMI: body mass index; ASA: American Society of Anesthesiologists; * median (range); † mean ± standard deviation.

## Data Availability

The datasets used and/or analyzed during the current study are available from the corresponding author on reasonable request.

## Supplementary Materials

The following supporting information can be downloaded at https://www.mdpi.com/article/10.3390/jcm15176736/s1. Supplementary File S1: PRISMA Checklist [46]; Table S1: GRADE evaluation for significant outcomes.
