# Supplementary material for: Hugo RAS Versus Da Vinci in Colorectal Surgery: A Systematic Review and Outcome Meta-Analysis
Source: J Clin Med. 2026 Aug 30;15(17):6736. doi: 10.3390/jcm15176736 (PMC13565984; doi:10.3390/jcm15176736)
Supplement: Supplementary file 1 [file jcm-15-06736-s001.zip › Supplementary Table S1.pdf]

Supplementary Table S1. GRADE evaluation for significant outcomes.

Outcomes	No. of studies	No. of included patients		MD [95 % CI]	Quality assessment					Quality
		Hugo RAS	Da Vinci		Risk of bias[a]	Inconsistency	Indirectness	Imprecision	Publication bias	
Duration of surgery	[6, 26, 27, 35, 36]	130	240	25.98 [6.35–45.62]	Serious	Not serious	No serious	No serious	NA	Low

CI: Confidence interval, NA: Not applicable, SMD: Standardized mean difference. [a] Risk of bias assessed using the ROBINS-I tool.
